# Supplementary material for: Perceived benefits of community-based TB preventive treatment in children in Uganda: “When she sees other children getting the same medication, she will feel not alone.”
Source: PLOS Glob Public Health. 2026 Apr 2;6(4):e0006206. doi: 10.1371/journal.pgph.0006206 (PMC13046145; doi:10.1371/journal.pgph.0006206)
Supplement: S1 Text — (DOCX) [file pgph.0006206.s001.docx]

**Feasibility and acceptability of a community-based Differentiated Service Delivery (DSD) strategy to increase initiation of TB preventive therapy TPT for children and youth.**

**Patient Qualitative Interview Guide – Children**

**Child’s Household ID:**

**Child’s SONET ID:**

**Introduction:**

Thank you so much for agreeing to participate in this study. As you know from our conversation when we were completing the consent form, the purpose of our overall study is to learn your opinions about the best way to deliver care for children on medicines that prevent tuberculosis. This medicine is for children who are not now sick from Tuberculosis but who have been exposed to the germ that causes TB and have a ‘sleeping’ tuberculosis. We are hoping to learn more about what things help children start and complete the preventative treatment, challenges they encounter, and your overall thoughts about the things that may influence parents or guardians to start or to avoid starting their children who are at risk of getting TB disease on drugs which prevent TB disease.

The information you share with us will provide us with insights on how best to use a community approach to promote the use of treatments that prevent people who have come into contact with the germ that causes TB from falling sick with TB disease. If there are any questions that you don’t want to answer, that is fine. And if you decide at any time that you want to stop the interview, that is fine too.

I’ll be recording the interview, and then we’ll transcribe it. The transcript will only be used for research purposes, to understand the perceptions of health care providers about this topic. Your name will not be linked to the interview, and whatever you say will not be shared with anyone else in the clinic: not the nurse, not the physicians, pharmacist, or other staff. Also, this information will not be shared with anyone in your family, or with any other person outside your family – it is totally confidential. This recording will be destroyed at the end of the study.

(TURN ON DIGITAL RECORDER)

Do you have any questions before we begin the interview?

I am (INTERVIEWER NAME) interviewing participant [PARTICIPANT#] on [DATE] [START TIME]

**1.General participant information**

I would like to start by asking personal questions about the community where [child’s name] lives:

- 1. How old is the child?
  2. Where does the child live?
  3. For how long has the child been staying there? Is this home for the child? Is there any other place this child calls home?
  4. Whom does the child stay with *(Probe for household structure and family composition)*? Tell me about who meets the day-to-day needs of this child and how? *(Probe for source of income and other forms of support).*
  5. What is the relationship between the child and the primary caregiver?
  6. What do you feel are the most pressing health care needs for this child?

**2. HIV care experiences – only applicable to patients who have self-reported or tested HIV positive (through SONET)**

1. Does the child have HIV?
   1. If yes, continue.
   2. If no, skip to next section ‘**TPT Knowledge**
2. If primary caregiver is the mother, ask; what is your HIV status?
   1. Positive
   2. Negative
3. Now I would like to ask you some questions about your experiences with HIV care and treatment:
   1. How long has [child’s name] been going to the ART clinic?
   2. When was the last time this child came to the clinic to see a clinician? How was the care the child received?
   3. What is the care like in this clinic? (*Probe for provider attitudes, waiting times, drug availability, privacy and confidentiality, community-based ART delivery etc.*).
4. Perceptions about TB care in the clinic, and TB in the community:
5. What services does the HIV clinic offer for TB testing? Has your child ever been tested or screened for TB there?
6. What views/perceptions do you think people in this ART clinic have about TB? (probe for stigma, causation)
7. What perceptions do you think people in this ART clinic feel about TB among children?
8. Do you think your child is at risk for TB? Why or why not?
9. How do people in your community feel about children who are sick with TB?
10. Do you know children who are infected with TB in your community? (*They should not name them.)* How do you feel about these children? How do you feel about their families
11. Do you know some People Living with HIV in your community?
12. How has TB affected people living with HIV in the community where this child lives?

**TPT Knowledge**

General knowledge about TPT and thoughts on care delivery?

1. What does latent/ ‘sleeping TB’ mean to you? How do you think others in the community feel about sleeping TB and the need to seek prevention treatment yet it does not make the children feel sick?
   - Probe: Have you heard of ‘sleeping TB’ before this study?
2. Have you heard about any treatments that can be given to children to treat sleeping TB (make the sleeping TB more likely to stay asleep)? If so, what did you hear about them?
3. How do you feel about treatments given to prevent children and adults to prevent them from falling sick with TB? [probe for positive and negative feelings about TB preventive therapy]
4. How do you think these TB preventive treatments should be delivered to children who need them?
5. Has your child received TB preventive treatment before?

This study aims to see whether it is feasible and acceptable to start children on TB preventive therapy and encourage them to take their medicines through a community group, facilitated by a VHT. We shall enroll children up to 12 years who have come into contact with someone with TB disease, or who have sleeping TB into a community group where we shall teach them about TB in general, and TB prevention in children. We shall encourage all children who are either contacts of TB patients or have a positive QFT test for latent TB to link to the facility to start TPT, but once they initiate TPT, they will be followed up in the community adherence group with monthly phone calls from the VHT to check for side-effects, and a group meeting every 3 months. The VHT will also assist with the teaching, and linking all those who need to see a health worker to the clinic We hope that this community approach will increase initiation, adherence, and completion of TB preventive treatment in this age group. **This is what we are calling a “TPT differentiated Service delivery intervention”**. Now that I have shared what we are trying to achieve with you, I would like to ask your opinion about this approach.

**Characteristics of the intervention** (if the child is under 5 years of age, the respondent to these questions is the primary caretaker.)

1.Current Model: Let’s talk a little more about different ways to deliver TPT. Currently TPT is offered through the health center. A child must first be evaluated by a clinician and then they must go to the clinic every month to pick up refills and for an evaluation.

- 1. What are the things you like about this strategy or receiving TPT at the clinic only? (Potential probes: safety, provider counseling))
  2. What are the things that you do not like about this strategy as a caregiver? (Potential probes: what about transport?

2.Relative advantages and disadvantages of DSD models: Another way that TPT could be delivered is through community groups like the one I have described above. In this model- children who need TPT would have a clinic visit to start TPT and then refills would be picked up by a group member and delivered to community-adherence groups like the one I have described above..

1. Relative advantage:
2. Have you heard of community-adherence groups before? Tell me more about that.
3. How do you think this community-based TB prevention group model will make initiation of TB preventive treatment by children more convenient? (probe comparisons to clinic model)?
4. How do you think this community-based TB prevention group model will make initiation of TB preventive treatment by children less convenient? (probe comparisons to clinic model)?
5. What do you like about the community-based TB prevention group model?
6. What do you dislike about the community-based TB prevention group model?
7. Complexity:
8. What challenges do you foresee in staring children from households affected by HIV on TB preventive treatment through a community-based TB prevention group?
9. What do you envision a VHT doing to help start children on TB preventive treatment in community-based TB prevention groups?
10. How do you feel about involving VHTs in helping to start children on TB preventive treatment and to assist with community-based TB prevention groups?
11. Adaptability
    1. What other ideas or recommendations do you have about how TPT can be delivered in the community or about community groups? *(What have we overlooked?)*

Who else do you think should be involved in the delivery of TP prevention through community groups?

**Inner setting**

1. Compatibility:
2. How does your child currently receive healthcare?
3. How can we best integrate TPT and community groups into how your child currently receives health care?
4. For children with HIV: What recommendations do you have for TPT to easily be integrated into HIV care? (probes: ART delivery, integrating screening or treatment in the clinic)?

**Characteristics of individuals**

1. Knowledge and beliefs about the intervention:

How do you think your child may benefit from community-based groups for TPT delivery?

How do you think your child may not benefit from delivery of TB prevention treatment through a community-based TB prevention group?

Do you have knowledge or experience with other types of community health groups?

1. Self-Efficacy:

How will community groups promoting tb prevention change your ability to access TB preventive treatment for your child?

How confident do you feel that you could participate in the community group? Why or why not?

Do you have any other suggestions about providing TB preventive treatment in community-based groups?

Do you have any other comments/suggestions about TB in general?

Thank you so much for taking time to speak with me today.

**END**
