# Supplementary material for: Perceived benefits of community-based TB preventive treatment in children in Uganda: “When she sees other children getting the same medication, she will feel not alone.”
Source: PLOS Glob Public Health. 2026 Apr 2;6(4):e0006206. doi: 10.1371/journal.pgph.0006206 (PMC13046145; doi:10.1371/journal.pgph.0006206)
Supplement: S3 Text — (DOCX) [file pgph.0006206.s003.docx]

**Feasibility and acceptability of a community-based Differentiated Service Delivery (DSD) strategy to increase initiation of TB preventive therapy (TPT) for children and youth**

**Key Informant Guide - District managers/Health care providers**

**Study ID of health care provider:**

**Title/role:**

**Name of Interviewer:**

**Introduction:**

Thank you so much for agreeing to participate in this study. As you know from our conversation when we were completing the consent form, the overall purpose of our study is to assess the feasibility and acceptability of a Differentiated Service Delivery (providing health services in different ways to better match the needs of patients) intervention to increase uptake of TB Preventive Treatment (TPT) for children in the community. We would like to learn about your perspective and experiences you have had providing TB prevention services to children and adults at risk for active TB in your district.

The information you share with us will provide us with insights on how best to use a community approach to provide TB preventive treatment to children at risk of TB. If there are any questions that you don’t want to answer, that is fine. And if you decide at any time that you want to stop the interview, that is fine too, just let us know.

I’ll be recording the interview, and then we’ll transcribe it. The transcript will only be used for research purposes, to understand the perceptions of health care providers about this topic. Your name will not be linked to the interview, and whatever you say will not be shared with anyone else in the clinic. This recording will be destroyed at the end of the study.

(TURN ON DIGITAL RECORDER)

Do you have any questions before we begin the interview?

I am (INTERVIEWER NAME) interviewing participant [PARTICIPANT#] on [DATE] [START TIME]

1. Please describe for me your roles and responsibilities in the prevention of TB/HIV in your district/health facility relating to your current professional position.
   1. What has been the most difficult aspect of fulfilling this role?
   2. What has been enjoyable about this role?
2. Please describe for me your roles and responsibilities in the management of TB/HIV in your district/health facility relating to your current professional position.
   1. What has been the most difficult aspect of fulfilling this role?
   2. What has been enjoyable about this role?
3. I would like to ask your opinion about community-based TB prevention in your district/health facility/village:
   1. How are TB prevention services currently delivered, outside of health facilities, in your district/village?
   2. What have you seen that needs to be improved about the way TB prevention services are delivered in your district/village?
   3. What challenges do you encounter delivering TB prevention services in the community?
   4. What challenges have you encountered delivering TB prevention services in the community that are specific to children?
   5. What are successful things you have seen in the way TB prevention services are currently delivered in the community?
4. What strategies have been put in place to improve the way TB services are delivered in this district?
   1. Can you describe for me some of the innovations that you put in place or witnessed being implemented in the district to ensure that children and families at risk of TB disease in the community receive TB preventive treatment?
   2. Which innovations have worked? Why do you think?
   3. Which innovations have not worked well? Why do you think?
5. In your opinion, what additional data would be needed to make effective decisions about TB prevention for children in the district?
6. In your opinion, what would be the best way to provide TB prevention services to children who are exposed to TB in the community. How best do you think this can be done?

This study aims to increase TPT initiation using a community-based differentiated service delivery model. We shall enroll children up to 12 years who are exposed to TB or who have latent TB into a community adherence group facilitated by a VHT and provide education about TB disease in general, and TB prevention in children specifically. We shall encourage all children who are either contacts of TB patients or have a positive QFT test for latent TB to link to the facility to start TPT, but once they initiate TPT, they will be followed up in the **community adherence group** with **monthly phone calls from the VHT**, and a **group meeting every 3 months.** We hope that this community approach will increase initiation, adherence, and completion of TB preventive treatment in this age group. **This is what we are calling a “TPT Differentiated Service Delivery (DSD) intervention”**. Now that I have shared what we are trying to achieve with you, I would like to ask your opinion about this approach.

1. Relative advantage:
   1. How do you think a TPT DSD intervention will make initiation of children on TB prevention treatment more/less convenient for healthcare workers/VHTs in your district?
   2. What specific challenges do you foresee with this approach? Tell me more about that.
   3. What would need to be in place first to facilitate this approach? Tell me more about that.
   4. What would be helpful to sustain this approach over time? Are there adaptations you would recommend?
   5. How do you think this TPT DSD intervention will make initiation of children on TB prevention treatment more/less convenient for households affected by HIV in particular?
2. Complexity:
   1. What specific challenges do you foresee for parents/guardians who start their children on TB preventive treatment within a community-based group with the help of a VHT compared to receiving this treatment from the health facility?
3. Adaptability:
   1. What would make it easier to adapt this intervention [*describe*] to meet your local needs as a health facility/district or a community?
   2. What would help your facility to adapt to this change?

**Inner setting**

1. Compatibility:
   1. How well is the community-based TPT DSD approach likely to fit into the current TB clinic structure? What tensions will it produce among the personnel involved? What features of TPT DSD do you think would cause the most problems?

For VHT only:

1. How well is the community-based TPT DSD approach likely to fit into your daily TB work as a VHT? What tensions do you foresee in working with the health facility to implement this approach?
2. Leadership engagement:
   1. In what ways would you be able (or willing) to support the implementation of this community-based TPT DSD intervention in your health facility/district?
   2. What constraints (time, money, space) might make it difficult to support, and why?

**Characteristics of individuals**

1. Knowledge and beliefs about the intervention:
   1. What is your opinion about the effectiveness of TB Preventive Therapy in preventing children from getting active TB disease?
   2. What is your opinion about DSD models? What do you know about them?
   3. What would you imagine parents believe about community-based delivery of TB preventive therapy for their children?
   4. Do you have experience with other types of DSD for other conditions?
2. Self-Efficacy:
   1. How might community-based TPT DSD groups change patients’ ability to access TB preventive treatment for their children?
   2. How confident are you in your ability to support this type of TPT DSD? Why or why not?
3. What do you imagine fellow health care workers would feel about supporting community based TPT models?

What additional thoughts do you have about using community-based DSD interventions to improve the uptake of TB preventive treatment in children and families at risk of TB?

**END**
