# Supplementary material for: Perceived benefits of community-based TB preventive treatment in children in Uganda: “When she sees other children getting the same medication, she will feel not alone.”
Source: PLOS Glob Public Health. 2026 Apr 2;6(4):e0006206. doi: 10.1371/journal.pgph.0006206 (PMC13046145; doi:10.1371/journal.pgph.0006206)
Supplement: S1 Data — (PDF) [file pgph.0006206.s004.pdf]

Table S1: Qualitative data codebook

Participant Group	Parent code	Child code	Code Definition
**Clients only**	**01. Personal characteristics of pts**	01.01. Personal context	Age, place of residence, duration of residence, HH composition
		01.02. Child's needs	(Self-explanatory -- can go beyond TB to include nutrition, etc)
	**02. HIV care experiences**	02.01. ART experience	Duration on ART, ART adherence challenges and facilitators
	includes HIV status of mother and child	02.02. Perceptions of care	Experiences with HIV clinic, last clinic visit, gernal perceptions of care
	**03. Perceptions about TB care & prevention**	03.01. Knowledge of services	includes knowledge of services offered
		03.02. TB risk perceptions & beliefs about cause	What they perceive about TB including risk factors. Why or why not?
		03.03. TPT Knowledge & perceptions	Includes own and community's knowledge of latent TB, medication to treat latent TB, feelings about TPT; includes perceptions of risks and benefits
	**04. Perceptions about people with TB and HIV**	04.01. Perceptions about people with TB disease	includes community and own perceptions about children who are sick with TB and their families.
		04.02. Perceptions about PLHIV	includes perceptions about how TB affects PLHIV in the community
		04.03. Social intersection of HIV & TB	Includes views/perceptions of PLHIV about TB in general, and in older children including stigma and causation. (Use descriptors to determine whether the interviewee is HIV-positive or -negative)
		04.04. Stigma (re people or services)	Stigma about HIV, TB, or treatment services
Clients, District managers and health care providers, VHTs	**05. Provider roles and resposibilities**	05.01. TB/HIV specific responsibilities	Any roles and responbilities related to management and prevention of TB (make sure to code HIV and TB in distinct blocks, not altogether as one highlighted chunk)
		05.02. Difficult aspects of TB/ HIV care	This includes any difficult aspects about fulfiing their professional roles
		05.03. Enjoyable aspects of TB/HIV care	This includes any enjoyable aspects of fulfilling this role
		05.04 VHT Specific Roles	Includes perceptions of VHT work beyond TB and HIV, such as immunizations, hygiene, sanitation, preventive care. Can also include HIV and TPT care.
	**06. Delivery of TB prevention/management services**	06.01. Current practices	This includes opnions on how CB-TB prevention services are delivered out of health facility/community
		06.02. General service delivery challenges	This includes general challeneges encountered during delivery of TB prevention services in the community. This can be double coded with challenges faced by VHTs
		06.03. Delivery successes	Any description of the successsful aspects of TB delivery in the community
		06.04. Pediatric TPT / TB care challenges	This includes challneges encountered during delivery of TB prevention services in the community for children; includes not having child friendly formulas, x-ray challenges
		06.05. VHT specific challenges	This includes general challeneges encountered during delivery of TB prevention services in the community by VHT.
	**07. Perceptions about service delivery**	07.01. Knowledge and beliefs about the delivery models	This includes opinions of TPT in preventing active TB in children, opinons about DSD models (own or other persons), expereinces with other types of DSD models for other conditions

Provider specific		07.02. Self-efficacy to deliver or participate in DSD models	Own or other's beliefs in the ability to support TPT DSD approaches.
	**08. Current & innovative strategies for TB care**	08.01.Current strategies	Current strategies in place whether they have been determined to be successful yet or not.
	This includes descriptions on the strategies that have been put in place to improve the way TB services are delivered in the district	08.02. Successful innovations	Includes innovations that have worked and why.
	Includes descriptions of innovations in place to ensure that children and families at risk of TB in the community receive TPT.	08.03. Unsuccessful innovations	Includes innovations that have nit worked and why.
	**09. Percieved benefits of CB-DSD TPT**	09.01. CB Self efficacy	Includes descriptions on how community-based TB prevention groups might change patients' ability to access TB preventive treatment for their children, and
		09.02. VHT optimisation	includes how TPT models could improve VHTs ability to support community based TB prevention, how CB might make VHT work easier
		09.03. Improved access (general)	Includes descriptions of how community based TPT delivery may improve access to TPT for children (use descriptors to sort for households affected by HIV)
		09.05. Other CB-DSD benefits	
	**10. Perceived barriers**	10.01. TPT initiation inTPT-DSD barriers	includes descriptions on challenges of using a TPT-DSD model for TPT initiation from the perspective of health workers/VHTs or patients
		10.02. Provider adaptability barriers	Includes descriptions on what would make it challenging to adapt this intervention to meet community needs, and what would make it difficult for VHTs/health care workers and managers to adapt to community based TB prevention.
		10.03. VHT/ Facility compatibility	Includes descriptions of tensions that may arise between VHTs and the health facility while implementing this approach.
		10.04. Constraints (logistical / material / mo	Includes constraints (time, money, space) that might make it difficult to get support for TB prevention and why. Includes Leadership engagement and support
		10.05. Other perceived barriers	Includes peoples' negative feelings about TB preventive teatment
	**11. Perceived facilitators**	11.01. TPT initiation in TPT-DSD facilitators	Includes descriptions on how the TPT-DSD model will make TPT initiation easier for health workers/VHTs or patients
		11.02. Facilitate provider adaptability	Includes descriptions on what would make it easier to adapt to adapt this intervention to meet community needs, and what would make it easier for VHTs/health care workers and managers to adapt to community based TB prevention.
		11.03. VHT/ Faciltiy Compatibility facilitators	Inludes descriptons on how well the community-based TB prevention group approach is likely to fit into the daily work of a VHT/health care worker or manager
		11.04. Leadership engagement and support	Includes descriptions on ways VHTs/health care workers would want to be supported to implement community based TB prevention
		11.05. Other perceived facilitators	Includes people positive feelings about TB preventive treatments
	**12.Recommendations**	12.01. Delivery TPT services-district/village level	discussions about how to improve uptake of TB preventive treatments using community based interventions.

		12.02. Delivery TPT services-children	Includes discussions about how TPT should be delivered to children
		12.03. Needs	Additional data, facility needs
		12.04. Sustainability	Any discussions on best ways to sustain the TPT-DSD approach and recommendations on adaptions.
	**13. Good Quote**	13.01. Quotable Quotes	
		13.02. Translation Check	
		13.03. Policy Implication	
