# Supplementary material for: Perceived benefits of community-based TB preventive treatment in children in Uganda: “When she sees other children getting the same medication, she will feel not alone.”
Source: PLOS Glob Public Health. 2026 Apr 2;6(4):e0006206. doi: 10.1371/journal.pgph.0006206 (PMC13046145; doi:10.1371/journal.pgph.0006206)
Supplement: Attachment — Submitted filename: SONET Qual paper_ Response to reviewers v17 Feb 2026.docx [file pgph.0006206.s007.docx]

5^th^ February, 2026

Response to reviewers

Dear Editor,

We thank the reviewers for their very helpful comments. In response to the reviewers’ remarks, we have now included a data availability statement. The raw data cannot ethically be made publicly available because it contains patient details that may inadvertently reveal their identities. In addition, we have explained how individual researchers can request the data through an independent scientific review committee, expanded our description of the sampling approach, added a table describing the study participants, and included a limitations section in the manuscript. We think this has strengthened the paper and appreciate the review of our submission. Below is our point-by-point response to the reviewers' and editor’s comments, which are in italics.

Sincerely,

Dr. Elijah Kakande

**Editor’s comments**

**Please include a complete copy of PLOS’ questionnaire on inclusivity in global research in your revised manuscript.**

Response: We now include a complete copy of the journal’s questionnaire on inclusivity to the submission.

**Review Comments to the Author

Please use the space provided to explain your answers to the questions above. You may also include additional comments for the author, including concerns about dual publication, research ethics, or publication ethics. (Please upload your review as an attachment if it exceeds 20,000 characters)**

**Reviewer #1: With interest I have read this well-written manuscript about an important topic**

**1. Line 179 – Perspective in quotation. The quoted text uses a female pronoun (“she”), while the description indicates it refers to a male child. This creates ambiguity about whose perspective is represented—the son, daughter, or mother. Please clarify.**

Response: We appreciate the reviewer's observation. The study participant was indeed female. We have now corrected the description, and it now reads; “*Female caregiver of a 6-year-old female child” (line 196)*

**2. A limitation of the study is that program manager perspectives were not included. Incorporating these could provide valuable insights into the sustainability of this community-based intervention. Any discussion on sustainability would strengthen the manuscript.**

Response: We appreciate the reviewer’s comment. We agree that the perspective of a program manager from the Ministry of Health would enrich the paper. However, while we did not interview managers at the Ministry of Health level, we included the perspectives of two high-level program managers/program implementers at the district level. Specifically, we included the District Health Officer (DHO), who oversees health services for over 100,000 people in his administrative unit and the district TB focal person who serves on the District Health Team, a committee in charge of overseeing implementation of Ministry of Health and specifically TB policies at the district level. These perspectives offer insights into the potential sustainability of the proposed approach. We now include a description of study participants in the results section which now reads, *“The health care providers included 3 health managers (District Health Officer, District TB focal person, and Health facility In-charge)”. (lines 177-178)*

To further address the reviewers’ concern, we have included a statement in the limitations section of the paper, which now reads: *“Second, we did not explore the perspectives of managers at the level of the Ministry of Health, which would expand our understanding of the feasibility and sustainability of CAGs for TB prevention in children. However, we included the perspectives of the District Health Officer and District TB focal person who oversee the implementation of TB guidelines provided by the Ministry of Health in their district to provide insights into the sustainability of this approach” (lines 450-453)*

**3. Methods: The methods section needs more detail on the sampling approach. The authors mention a purposive sample, but the criteria for selection are not specified. Please elaborate on the basis for participant inclusion.**

Response: We appreciate the reviewer's comment. Regarding our study sampling, our focus was on children diagnosed with latent TB infection after one year of follow-up in the SONET study, as these would be the intended beneficiaries of a TB prevention community adherence group. Our sample was enriched for this category (7/10). We also included some caregivers of children living with HIV to capture their perspectives as well (3/10). To capture the perspectives of lay providers, we selected a sub-sample of Community Health Workers in the study catchment area. Finally, we selected a sample of health workers involved in the provision of TB care at the health facility providing TB care in the study area, including some with oversight roles at facility and district levels.

We have now revised this section in the manuscript and it reads as follows; *“Between 15th September and 12th October 2023, a trained team of qualitative researchers administered in-depth, semi-structured interviews to a purposive sample of; 1) Caregivers of children (n=10) participating in the SONET study who also had a diagnosis of latent TB infection after 1 year of follow-up, or who were living with HIV (these groups were purposefully selected because they would be the intended beneficiaries of TPT in CAGs) 2) CHWs (n=4) who deliver services to households in participating villages, and health care providers (n=6) including doctors, clinical officers, and nurses involved in the management and delivery of TB services at the public health facility within the study catchment area” (line 132-140).*

**4. Demographic table. Include a table summarizing demographic characteristics of the interviewees. For CHWs and HCWs, it would be particularly useful to report their training background and tenure in their current roles.**

Response: We appreciate the reviewer’s comment. We now include a table describing participant demographics, including the cadre and sex (Table 1 below). We agree that data on training background and tenure in current roles would have improved our understanding of who participated; however, we did not collect these data and are unable to include them in Table 1.

| **Cadre** | **Male** | **Female** | **N= 20** |
| --- | --- | --- | --- |
| Health care providers (including 3 health managers) | 1 | 5 | 6 |
| Community Health Workers | 2 | 2 | 4 |
| Caregivers of children exposed to TB | 2 | 8 | 10 |
| Children whose caregivers were interviewed  Diagnosed with Latent TB  Children living with HIV | 3  2 | 4  1 | 7  3 |

**5. Appears to be an error with heading “1.2.3,” which is used twice. Please correct this duplication.**

Response: We appreciate the reviewer for this comment. We have corrected this error.

**6. Sample size and data saturation. The sample size is small, and the manuscript does not address data saturation. This raises questions about the representativeness of the findings. It seems unlikely that saturation was achieved, especially with only four CHWs interviewed. Please discuss this limitation.**

Response: We thank the reviewer for this insightful comment. Data saturation was assessed at the level of the whole sample, rather than sub-groups. We now include a statement in the methods section to improve clarity, and it now reads; *“Thematic saturation was assessed at the level of the overall dataset rather than within individual participant subgroups. Subgroup sample sizes were not intended to achieve independent saturation; instead, input from health care providers, community health workers, and caregivers informed the identification and refinement of the core themes” (lines 140-144).*

To further respond to the reviewer’s concern, we now include this in the limitations section: *“As a pilot study, we kept our sample population small. We therefore do not claim data saturation for each subgroup within the sample (e.g. CHWs). However, we did strive to include participants from each of the main stakeholder groups: providers, caregivers, and CHWs" (lines 456-459).*

**7. Line 347 – Missing reference. Add a reference for the statement on line 347, including the trial’s results and why they are relevant in this context.**

Response: We acknowledge the reviewer’s comment. The cluster randomized trial evaluating community-based delivery of TPT at the household level is so important to the subject under discussion. We described the trial briefly and referenced it in the introduction of our paper, but now include it in the discussion section as well, and appropriately referenced it.

*In a cluster randomized trial conducted in Uganda and Cameroon, CHWs participated in TB screening and follow-up of people taking TPT medication to great effect, leading to increased contact tracing coverage and TPT completion compared to clinic-based TPT delivery [9] (lines 371-374).*

*Reference: Bonnet M, Vasiliu A, Tchounga BK, Cuer B, Fielding K, Ssekyanzi B, et al. Effectiveness of a community-based approach for the investigation and management of children with household tuberculosis contact in Cameroon and Uganda: a cluster-randomised trial. The Lancet Global Health. 2023;11(12):e1911-e21.*

**8. Line 402 – Government proposal. Provide a reference to support the statement that the government is proposing CHEWs.**

Response: We thank the reviewer for this comment. We have provided a reference to this statement in line 431.

*One of the solutions proposed by the government is the creation of community health extension workers (CHEWs) [40], a new cadre of community health workers with a higher level of education, more extensive training, and some remuneration, who will provide support and supervision to groups of community health workers at the village level (lines 436-439).*

*Reference: O’Donovan J, Stiles CE, Sekimpi D, Ddumba I, Winters N, O’Neil Jr E. Potential challenges of implementing the Community Health Extension Worker programme in Uganda. BMJ global health. 2018;3(4).*

**9. Discussion: The limitations of this study should be explicitly discussed in the discussion section.**

We thank the reviewer for this comment. We now include a section on study limitations as described below;

*Our study has some limitations. First, the study explored the feasibility of a hypothetical community-based TB program, seeking to understand barriers to TPT implementation using CAGs for children prior to actual implementation. While this is highly informative and could aid the development of a sustainable and highly effective program, it may not reflect actual barriers that people will face during the implementation of such a program. Second, we did not explore the perspectives of managers at the level of the Ministry of Health, which would have expanded our understanding of the feasibility and sustainability of CAGs for TB prevention in children. However, we included the perspectives of the District Health Officer and District TB focal person who oversee the implementation of TB guidelines provided by the Ministry of Health in their district to provide insights into the sustainability of this approach. Finally, as a pilot study, we kept our sample population small. We therefore do not claim data saturation for each subgroup within the sample (e.g. CHWs). However, we did strive to include participants from each of the main stakeholder groups: providers, caregivers, and CHWs” (lines 446-459)*

**10. Conclusion: In the conclusion, emphasize the importance of a tailored approach that meets individual preferences.**

Response: We agree with the reviewer on the importance of person-centered approaches and have expanded the conclusion to emphasize this important point:

*Additionally, TPT delivery for children in a community setting will require a person-centered approach that meets individual preferences and needs (lines 472-473).*

***Reviewer #2: Review of Kakande et al.*** *This qualitative study reports on findings from in-depth interviews of caregivers and health care workers about a proposed community-based delivery model for TPT that includes adherence groups for support. The stakeholder perspectives are valuable, but the manuscript is unclear about how the proposed model of TPT delivery that was described to participants may have differed from the model they experienced.*

Response: We appreciate the reviewer for this thoughtful review of our work.

**Major
Introduction
1. Lines 96-99 indicate that “community adherence groups focusing on children where TPT is delivered within the group by a CHW with healthcare provider support can overcome practical barriers of clinic-based TPT…”. What is the need for the present study if this evidence is already known? The reference cited seems to be about active case finding rather than community adherence groups for TPT.**

Response: We thank the reviewer for this comment. While the benefit of community adherence groups has been well studied in HIV care, particularly in adults, the benefits of this strategy for TB preventive treatment are less well described. To our knowledge, we are the first to describe the potential benefits of a CAG for TB preventive treatment in children. We agree with the reviewer that the reference included was not appropriate. We now include the correct reference (line 100) to these statements;

*Pellecchia U, Baert S, Nundwe S, Bwanali A, Zamadenga B, Metcalf CA, Bygrave H, Daho S, Ohler L, Chibwandira B, Kanyimbo K. “We are part of a family”. Benefits and limitations of community ART groups (CAGs) in Thyolo, Malawi: a qualitative study. Journal of the International AIDS Society. 2017;20(1):21374*

**Methods
2. Line 132 - The description reports purposive sampling. What criteria were used? How representative were the study participants of the stakeholder population?**

We appreciate the reviewer’s comment. Our target population was all caregivers of children with latent TB infection participating in the Socio-spatial Networks for TB infection among children and youth in rural Uganda (SONET), an epidemiological study that aimed to ascertain when and where children and youth acquire TB infection. The study screened all children aged 1-17 living in households in 25 villages in Ndeija subcounty in south-western Uganda (N=5879) for latent TB infection using QuantiFERON Gold plus (QFT), a blood-based assay (lines 121-124).

Regarding our study sampling, our focus was on children diagnosed with latent TB infection after one year of follow-up in the SONET study, as these would be the intended beneficiaries of a TB prevention community adherence group. Our sample was enriched for this category (7/10). We also included some caregivers of children living with HIV to capture their perspectives as well (3/10)(Table 1). To capture the perspectives of lay providers, we selected a sub-sample of Community Health Workers in the study catchment area. Finally, we selected a sample of health workers involved in the provision of TB care at the health facility providing TB care in the study area, including some with oversight roles at facility and district levels.

We have now revised this section in the manuscript and it reads as follows; *“Between 15th September and 12th October 2023, a trained team of qualitative researchers administered in-depth, semi-structured interviews to a purposive sample of; 1) Caregivers of children (n=10) participating in the SONET study who also had a diagnosis of latent TB infection after 1 year of follow-up, or who were living with HIV (these groups were purposefully selected because they would be the intended beneficiaries of TPT in CAGs) 2) CHWs (n=4) who deliver services to households in participating villages, and health care providers (n=6) including doctors, clinical officers, and nurses involved in the management and delivery of TB services at the public health facility within the study catchment area” (line 132-140).*

**3. Lines 140 – 145 describe three components of a differentiated service delivery model. It is unclear if the study participants experienced a service delivery model that included these components, or if they were presented as a hypothetical delivery model. Additionally, are the three components intended to represent alternative delivery models or parts of the same delivery model? How do parts 1 and 3 fit in the same model?**

Response: We thank the reviewer for this comment. The goal of this study was to understand the preferences of potential beneficiaries of this TB preventive model to allow for modifications prior to implementation. We agree that, as stated, the statement may be confusing. To improve clarity, we have revised the statement to explicitly state that this was a hypothetical model. The statement now reads: *The hypothetical differentiated service delivery model that we discussed with participants comprised three core components: 1) initial screening and initiation of TPT at the nearest public health facility, 2) Community Health Worker (CHW) led TB education in a CAG (where child TB contacts and their caregivers from households overseen by a CHW gather every 2-3 months at a community venue in their village for TB prevention services) and 3) CHW facilitated delivery of TPT in a CAG” (lines 149-154).*

**Results
4. Line 241 – The finding is that TPT delivery is dependent on a constant drug supply. How is this specific to the care model being evaluated since clinic-based delivery would also require that drugs are available?**

We appreciate the reviewer’s comment. Indeed, some of the perspectives of the persons interviewed may be cross-cutting, for example, the need for a constant drug supply. We have included these statements in our discussion section in response to the reviewer’s comment:

*“While the community CAGs are responsive to human resource deficits, they could be negatively impacted by drug stockouts and low funding. These system-level barriers, however, are not limited to CAGs and may impact other community-based approaches as well as clinic-based delivery, highlighting the need to strengthen the supply for TB commodities for successful implementation and scale up of TPT for children” (lines 413-418)*

**Discussion
5. Lines 309 – 312 – Stating that the participants perceived that a delivery model was beneficial makes it sound like they experienced this model and contributes to confusion about whether this model is hypothetical or has been implemented.**

We appreciate the reviewer’s comment. We have now revised the language to read as follows:

*In this qualitative study, healthcare providers, CHWs, and caregivers of children with TB infection felt that if implemented, a community-based delivery model with a CAG for TPT for children facilitated by a CHW would be beneficial for providing peer support for adherence. (lines 332-335).*

**Minor

6. CAG and community adherence group (spelled out) seem to be used at various points in the manuscript. I suggest using CAG after initial definition unless it is needed for clarity.**

Response: We appreciate the reviewer’s comment. We have now aligned the manuscript to reflect this.

**Abstract
7. Line 76 – The sentence ends during a phrase.**

Response: We appreciate the reviewer’s comment. We have now edited this to make the sentence complete. It now reads: *Implementation efforts for community-based TPT should proactively address these barriers to optimize delivery in rural communities in East Africa and similar settings (lines 75-77)*

**Introduction
8. Line 94 – “Barriers to TPT…” should cite the source of the information.
Methods**

Response: We thank the reviewer for this comment. We have included a reference to this information as below:

*Barriers to clinic-based TPT delivery include a lack of belief in efficacy and need for TPT, as well as practical barriers, including transport, long waiting hours at the clinic, and time constraints [7]. (lines 94-96)*

*Reference: Amuge PM, Ndekezi D, Mugerwa M, Bbuye D, Rutebarika DA, Kizza L, et al. Facilitators and barriers to initiating and completing tuberculosis preventive treatment among children and adolescents living with HIV in Uganda: a qualitative study of adolescents, caretakers and health workers. AIDS research and therapy. 2024;21(1):59.*

**9. Line 143 – define HH**

Response: We appreciate the reviewer’s comment. We now write HH in full as “households”.
*“Community Health Worker (CHW) led TB education in a CAG (where child TB contacts and their caregivers from households overseen by a CHW gather every 2-3 months at a community venue in their village for TB prevention services…….” (line 152)*

**Results
10. Line 200 – “One of the key…” is unclear if it refers to a finding from the current study or prior research.**

Response: We have now included a reference for this statement.

*One of the key barriers to facility-based TPT is the required investment in transport costs to sustain follow-up visits [7]. (lines 214-215)*

*Reference: Amuge PM, Ndekezi D, Mugerwa M, Bbuye D, Rutebarika DA, Kizza L, et al. Facilitators and barriers to initiating and completing tuberculosis preventive treatment among children and adolescents living with HIV in Uganda: a qualitative study of adolescents, caretakers and health workers. AIDS research and therapy. 2024;21(1):59.*

**11. Line 208 – What evidence supports the finding that community based TPT reduces provider workload at the health facility? This seems like an important finding from providers, but the only evidence cited is a finding from caregivers and not providers.**

Response: We agree with the reviewer that this is an important finding. Indeed this was a view shared by healthcare providers as well. We now include a quote to support this view:

*“It will [be convenient] because in one way or the other this approach will be helping them, since VHTs will be reducing their work load at the health facilities, we have been receiving many children for these refills but if they can now see that, there is now someone who can take for them the drugs to these children, they would have relieved them on their work load at the facility, and they will even have the good outcomes for those children who are initiated on the TPT getting their refills from the community.” – Female health care worker (lines 234-240)*
 **Discussion
12. Line 332 – The sentence is incomplete.**

Response: We thank the reviewer for this comment. We have now removed the incomplete statement from the manuscript, as this important work by Amuge and colleagues is discussed extensively elsewhere in the discussion section:

*“Amuge and colleagues identified individual-level barriers to TPT initiation among children and adolescents, including carers lacking time to take children to the health facility, perception that children shouldn't swallow drugs when they are not ill, and stigma [7]. However, this study was conducted among people with HIV in a clinic-based model, and while some barriers identified (such as stigma) may be similar in both clinic and community settings, others may not be generalizable to different contexts (such as the community setting) and populations (such as children without HIV). Our study expands our understanding of barriers to TPT implementation to children beyond the health facility” (lines 420-427).*

**13. Line 339 – The sentence is incomplete.**

Response: We have reviewed line 339, and it seems complete. We kindly request clarification from the reviewer about this. Here is the original statement.

*To our knowledge, this is the first study to explore a CAG for TPT for children, and our data suggest it may be acceptable and warrants additional study (lines 38-340).*
